# Diseases of the Heart and Circulation

**Published:** 1901-10-12

**Authors:** 


### Diseases of the Heart and Circulation.

(*Continued from page* 16.)

**Tobacco Heart.**—The uncomplicated effects of the over-use of tobacco in young men are palpitation in every case, in half the cases post-sternal oppression and pain, and exceptionally either angina or uncomfortable sensations in the left arm. Faintness or actual fainting occurred in a third of the cases. The heart is of ordinary size in half the cases, in a few it is very slightly enlarged, the impulse is often weak, and the pulse tension low. No cardiac murmurs are present. In men over 40 years of age suffering from the effects of tobacco, whilst palpitation is still the common complaint, pain, including angina, is put forward more prominently, and so are faints, faintness, and a feeling of impending death with a sense of cardiac irregularity. The heart is more frequently found to be large and feeble, with occasionally a weak mitral systolic murmur, the radial pulse is often irregular and the vessel wall thick. All the alarming symptoms pass away on leaving off the tobacco. Thus, precordial pain, angina, faintness and regular pulse in a man of 60 are not to be hastily regarded as evidences of grave disease, without further inquiry as to his habits.

**The Heart in Alcoholism** presents clinical characters as a whole very different from those of the tobacco heart ; there is generally evidence of actual pathological change in the size of the heart and the condition of the myocardium. The heart is always enlarged, the precordial impulse is weak, the sounds weak, and in a fifth of the cases apical systolic murmur is present. The action is irregular and accelerated, the pulse tension is low, and the radial artery often sclerosed. The cause of alcoholic heart often becomes affected by the occurrence of cirrhosis of the liver or Bright's disease.

In gouty subjects who come under observation for cardiac trouble the heart is generally enlarged with feeble action, but regular and of normal rate ; in half the cases an aortic systolic murmur is present. A high-tension pulse and distinct thickening of the arterial walls is generally found. The patients mostly complain of precordial pain, and in a fourth of the cases the pain is anginal, often of a most pronounced type.

**Strain.**—People who strain their hearts after middle life chiefly complain of precordial oppression, a sense of palpitation, and irregular action and pain, which may amount to angina ; whilst the heart is found enlarged, with a feeble, regular pulse and precordial impulse, and but rarely any abnormal cardiac sounds. Such a condition is apt to be found in a man of, say, 50, who is attempting to resume the athletic exercises of his youth in order to reduce his weight, relieve his liver, or dispel gout.

**Syphilis** appears to account for a considerable proportion of the more serious cases of heart disease in older people, and this disease is often endo- as well as myo-cardial; many have double aortic disease, others a loud, ringing second sound over the aortic area. Angina is the most frequent complaint, and sudden death is common. In the diagnosis of any particular case of heart failure in middle later life, we have thus to deal with strain, gout, syphilis, tobacco, an old rheumatic lesion, or a combination of two or more of these; and, as a rule, inquiry will elicit which of these factors has been at work. Physical examination follows. In interpreting the physical signs it should be remembered that a mitral presystolic murmur is never significant of a senile lesion. Again, an aortic diastolic murmur, whether alone or with an aortic systolic murmur, means something more than atheroma, tobacco and alcohol; it is a result of syphilis or of acute or chronic valvular strain. It is always grave, being so frequently associated with coronary disease and consequent myocardial failure. An aortic systolic murmur is a common sign of atheroma of the aortic arch and valves, in association with regular or irregular gout, diabetes or corpulence; or it may be due to syphilitic disease. An ill-developed basic systolic murmur is not uncommon in alcoholism, chronic Bright's, and nervous strain, but is difficult to dissociate from anæmia. A fully-developed systolic mitral murmur is usually traceable to endocarditis of rheumatic or other origin, but in some cases is due to valvular atheroma caused by gout. An ill-developed mitral murmur is not of value in differential diagnosis, as it signifies merely relaxation of the left ventricle. An accentuated aortic second sound is of value in the diagnosis of arterial tension and atheroma. A definite doubling of the first sound followed by an accentuated second sound is practically pathognomic of Bright's disease. A normally sized heart with irregularity, increased frequency and a variable systolic murmur in the mitral area is characteristic of tobacco poisoning. A heart enlarged on both sides, and acting irregularly without failure is, apart from cardiac failure, suggestive of strain in early life. The prognosis of tobacco poisoning is favourable; as a rule improvement begins early and recovery is complete. The prospect of life in the alcoholic heart is most unfavourable; for as a rule the habit is too firmly established to be given up, and other organs besides the heart are affected. Occasionally the end is sudden from fatty degeneration, otherwise by slow cardiac failure and dropsy. The prognosis in gouty heart is difficult; in many cases arrest of degeneration takes place sometimes for very many years. In old gouty subjects sudden death may occur, not from the lesion of which a murmur is the sign, but from associated coronary atheroma. In syphilitic lesions of the heart and vessels prognosis is very bad; only about a half improved under treatment, and a fifth died within a few years. In cases of muscular strain, whether this took place in youth or after 40 years of age, the prognosis is as a rule good if the patient refrains from exertion and is content to live an invalid life. An etiological standpoint in a survey of the disorders of the heart in advanced life is thus of great service in prognosis, and it enables us to pursue a rational mode of treatment. It should not be forgotten, too, that a large proportion of the distress, dangers, and disabilities attending degeneration of the heart are due to some additional or extrinsic disturbance—distension of the stomach, constipation, worry, or exertion—which alone, not the pathological condition, calls for immediate therapeutical attention. Many of the unfavourable influences can be avoided by simple exercise of the will—for instance tobacco, alcohol, tea. Gout calls for temperance, not only in drinking, but in eating also; whatever they eat, it should be little, for moderation in amount is far more important than avoidance of the theoretical antecedents of uric acid. Ill-considered attempts at athleticism after the age of 40 should be prevented. In syphilitic lesions iodide of potassium should be given freely, whilst in atheroma iodide and arsenic are useful. But the main indication is to sustain the nutrition of the myocardium, by cardiac stimulants and tonics, hœmatinics, stomachics and laxatives, by the control of nervous influences, and by the use of active and passive exercises and baths. When true failure occurs there are three great therapeutical indications—to reduce the peripheral resistance, to increase the vigour of ventricular contraction, and to rehabilitate hypertrophy. Bodily rest, a light solid diet, a definite allowance of alcohol, active purgation with mercurial salines and jalap, and the exhibition of sufficiently large doses of digitalis with salines and other diuretics.

